# Extracorporeal life support and systemic inflammation

**DOI:** 10.1186/s40635-019-0249-y

**Published:** 2019-07-25

**Authors:** Abdulrahman Al-Fares, Tommaso Pettenuzzo, Lorenzo Del Sorbo

**Affiliations:** 1 Adult Critical Care Medicine Fellowship Program, University of Toronto, Toronto, Canada; 2 Al-Amiri Hospital, Ministry of Health, Kuwait City, Kuwait; 3 Interdepartmental Division of Critical Care Medicine, Toronto General Hospital, University of Toronto, Toronto, Canada; 4 Toronto General Hospital, 585 University Avenue, PMB 11-122, Toronto, Ontario M5G 2 N2 Canada

**Keywords:** Inflammation, Biomarkers, Cytokines, Extracorporeal life support, Extracorporeal membrane oxygenation, Extracorporeal carbon dioxide removal, Cardiopulmonary bypass

## Abstract

Extracorporeal life support (ECLS) encompasses a wide range of extracorporeal modalities that offer short- and intermediate-term mechanical support to the failing heart or lung. Apart from the daily use of cardiopulmonary bypass (CPB) in the operating room, there has been a resurgence of interest and utilization of veno-arterial and veno-venous extracorporeal membrane oxygenation (VA- and VV-ECMO, respectively) and extracorporeal carbon dioxide removal (ECCO_2_R) in recent years. This might be attributed to the advancement in technology, nonetheless the morbidity and mortality associated with the clinical application of this technology is still significant. The initiation of ECLS triggers a systemic inflammatory response, which involves the activation of the coagulation cascade, complement systems, endothelial cells, leukocytes, and platelets, thus potentially contributing to morbidity and mortality. This is due to the release of cytokines and other biomarkers of inflammation, which have been associated with multiorgan dysfunction. On the other hand, ECLS can be utilized as a therapy to halt the inflammatory response associated with critical illness and ICU therapeutic intervention, such as facilitating ultra-protective mechanical ventilation. In addition to addressing the impact on outcome of the relationship between inflammation and ECLS, two different but complementary pathophysiological perspectives will be developed in this review: ECLS as the cause of inflammation and ECLS as the treatment of inflammation. This framework may be useful in guiding the development of novel therapeutic strategies to improve the outcome of critical illness.

## Background

Inflammation is a central facet in the complex pathophysiology of critical illness. Irrespective of cause, critical illness initiates the innate and adaptive immune systems, resulting in systemic inflammatory response syndrome (SIRS) [1–6]. Elevated levels of pro-inflammatory cytokines have been associated with mortality in trauma, complex surgical interventions, sepsis, adult respiratory distress syndrome (ARDS), and cardiogenic shock [7]. Additionally, anti-inflammatory response if unbalanced results in anergy and immunosuppression [8]. Furthermore, multiple organ failure has been postulated to be due to massive activation of inflammatory mediators by critical illness resulting in vascular endothelial damage, permeability edema, and impaired oxygen availability to mitochondria [9]. Following the inception of modern intensive care units (ICU), therapeutic interventions and life support strategies have led to significant reduction in inflammatory mediators and hence mortality [10–14].

Extracorporeal life support (ECLS) is a term than has been used interchangeably with extracorporeal membrane oxygenation (ECMO), but it encompasses all extracorporeal technologies, including cardiopulmonary bypass (CPB), ECMO in all its configurations, and extracorporeal carbon dioxide removal (ECCO_2_R). Since the success of CPB for short-term circulatory support in the 1950’s, enthusiasm has grown to translate this technology to intermediate and long-term support for critically ill patients [15]. The first report of the use of veno-arterial extracorporeal membrane oxygenation (VA-ECMO) for respiratory failure was two decades later [16]. Although initial randomized clinical trials failed to demonstrate any clinical benefit of this technique [17, 18], with the advancement in technology and improvement in the safety profile, a resurgence of ECMO have been seen in the last decade with an exponential expansion in the number of ECMO centers worldwide. Moreover, improvement in outcomes has also been reported with survival of 57% to hospital discharge for patients with respiratory failure and 41% to hospital discharge for patients with cardiac failure [19].

Although lifesaving in many situations, complications of ECLS, whether mechanical, pump related, secondary to bleeding, or infection, are common and often contribute to morbidity and mortality [15, 19]. One of the relevant complications of ECLS is the associated inflammatory response. A rapid rise in pro-inflammatory cytokines following initiation of ECLS [20–22] is thought to be associated with an innate immune response [23], which if severe may lead to end-organ dysfunction and death [24, 25].

It is challenging to discern the extent of the inflammatory response that is solely due to ECLS or due to critical illness, underlying disease or ICU therapeutic interventions including complex surgical procedures and mechanical ventilation (MV) [26–30]. Furthermore, while the potential mechanical and inflammatory injury caused by other means of life support such as MV is well recognized within the critical care community [31], the importance of the inflammation associated with the application of ECLS is less understood.

**Fig. 1:**
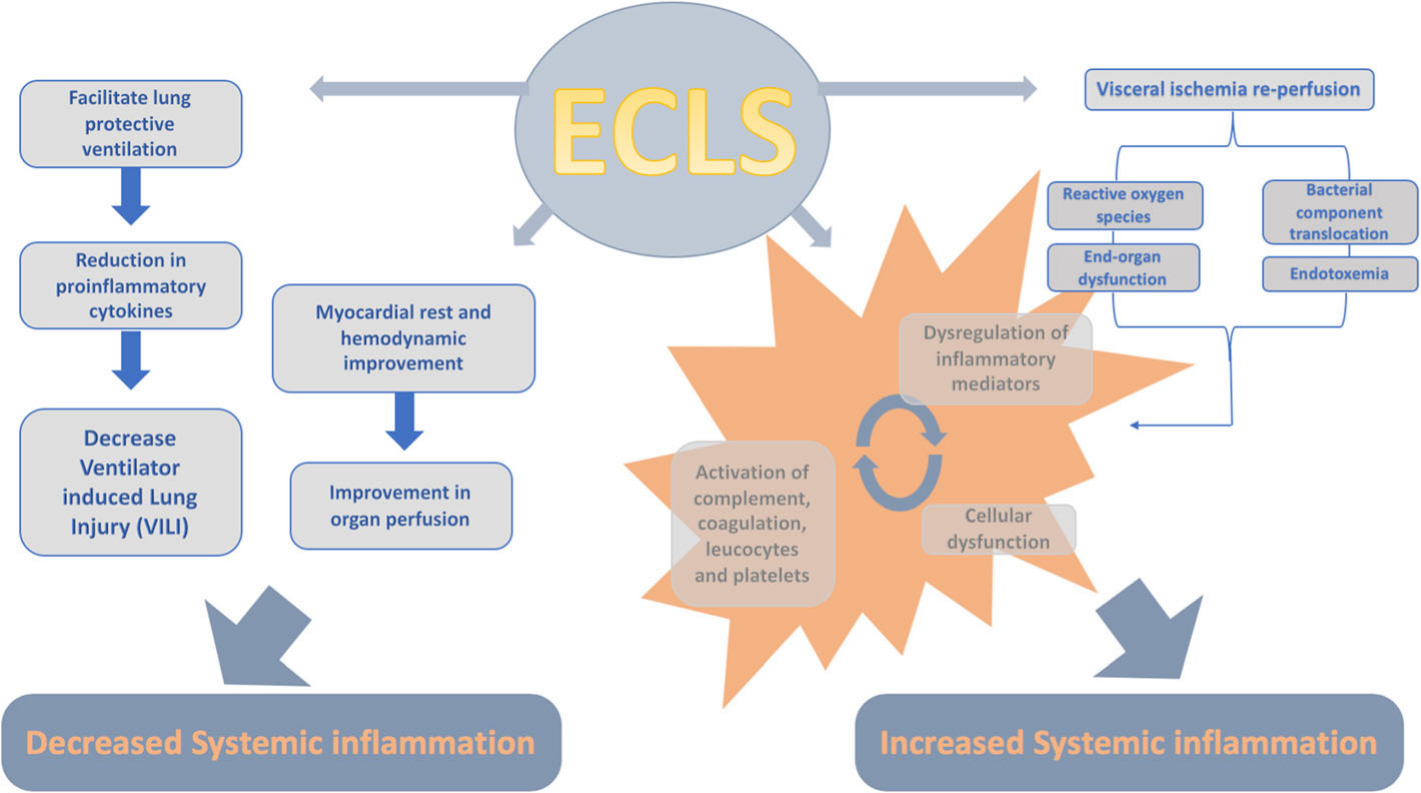
Pathophysiology ECLS and associated inflammatory response

In addition to addressing the impact on outcome of the relationship between inflammation and ECLS, two different but complementary pathophysiological perspectives will be developed in this review: ECLS as the cause of inflammation and ECLS as the treatment of inflammation (Fig. 1). This framework may be useful in guiding the development of novel therapeutic strategies to improve the outcome of critical illness.

## ECLS as a cause of systemic inflammation

Upon exposure of blood to the extracorporeal circuit during ECLS an inflammatory response might be triggered that mimics SIRS [32]. This is mediated by both humoral and cellular activation pathways, which are fundamentally interdependent but not fully understood during ECLS [33]. Most of the investigations on this specific issue have been developed with the CBP system, and hence a comprehensive evaluation is limited by the lack of rigorous studies on this issue conducted on ECMO and ECCO_2_R. Additionally, a majority of the studies are conducted in neonates and pediatrics and used an older technology, with less advanced pumps, circuits, and biocompatible materials, pre-dating modern ECLS. Therefore, the findings can be only prudently extended to all the more modern ECLS configurations and uses.

### Contact system, coagulation cascade, and complement

Following initiation of ECLS, the contact system becomes activated and subsequent byproducts of this activation promote coagulation and drive inflammation [33]. This activation process is rapid [34], resulting in neutrophil activation, release of nitric oxide and pro-inflammatory cytokines, as demonstrated during CPB [35, 36] and in neonatal ECMO [23]. The contact system activation triggers both intrinsic and extrinsic coagulation pathways, leading to clot formation and inflammation [33]. Notably, in simulated closed ECC, the expression of tissue factor (TF) by activated monocytes, or alternatively the TNF-α- and IL-6-induced release of soluble TF by endothelial cells, was evident without the need to be triggered by tissue injury and resulted in a 30-fold increase in thrombin formation [37, 38]. The complement system is also triggered upon initiation of ECLS [23, 39]. This mechanism is usually rapid with a peak in 1–2 h [23, 40–42] but is short lived and limited to 1–2 days following initiation of ECLS [40, 43]. This activation is mediated by C5a, C3a, C3b, and terminal complement complex and causes an increase in leukocyte recruitment, vascular permeability, and endothelial dysfunction [40, 43, 44].

### Role of platelets

Platelets are a major mediator of inflammation and not just hemostasis during ECLS [45]. Platelet activation has been extensively studied during CPB [46]. CPB causes structural and biochemical changes in platelets including differential expression of membrane receptors and formation of platelet conjugates due to shear forces created by circulatory pumps [47]. Less is known on the mechanisms of platelet activation during ECMO and ECCO_2_R. In one of these investigations in neonates supported with VV-ECMO for respiratory failure, platelets were found to adhere to the fibrinogen absorbed by the circuit, resulting in a time-dependent platelet activation along with persistent and progressive platelet dysfunction leading to the release of pro-inflammatory cytokines and expression of TF [48, 49].

### Role of the endothelium and leukocytes

Endothelial dysfunction in critical illness has been associated with poor outcomes [50] and plays also a major role in ECLS-induced inflammation. Alteration in endothelial cell gene expression occurs due to the effect of cytokines, complement, and reactive oxygen species, leading to pro-inflammatory mediators release and increased transmigration of leukocytes [48, 51]. The resulting neutrophil infiltration has been described to lead to ECLS-associated lung injury and end-organ damage [23–25, 52, 53]. Activation of neutrophils has been found in an experimental simulated ECC to be instantaneous [54], peaking within the first few hours of ECMO initiation and declining thereafter [55].

### Bacterial translocation

Other potential inflammatory mechanisms studied during ECLS are gut barrier dysfunction, bacterial translocation, and endotoxins release. During CPB and ECMO, endotoxins can be released in response to translocation of bacteria from ischemic gut mucosa into the blood stream [56, 57]. Lipopolysaccharide is released by Gram-negative bacteria and induces macrophages to release TNF-α and endothelial cells to release IL-6 [58]. Endotoxins stimulate circulating monocytes to produce cytokines, such as TNF-α [59] and blood-borne TF [60], thereby activating the coagulation cascade. Additionally, thrombin generation promotes inflammation, leading to a vicious circle.

### Human leukocyte antigen sensitization

Another interesting mechanism by which ECLS may promote inflammation is by triggering human leukocyte antigen (HLA) sensitization in subjects bridged to transplant with extracorporeal means of life support. HLA sensitization has been reported in pediatric patients supported with a ventricular assist device and ECMO while awaiting heart transplant [61, 62]. In addition, a recent report indicated that also patients bridged to lung transplant with ECMO might develop HLA sensitization [63]. However, the potential etiological mechanisms resulting into allosensitization during ECMO remain unclear and speculative.

### CBP-specific inflammatory response

CPB involves unique features that further contribute to inflammation (Table 1). The clamping of the aorta during surgery inflicts an ischemia-reperfusion injury to both the heart and the lungs and results in a significant inflammatory reaction [64]. Moreover, the protamine administered at the end of CPB for heparin reversal results in protamine-heparin complexes that are known to exacerbate the inflammatory response via activation of the classical and lectin complement pathways [65]. Furthermore, hemodilution can be employed in CPB and could lead to increased neutrophil activation and therefore SIRS [66, 67]. Finally, surgical trauma and the presence of blood-air interface due to cardiotomy suctioning, venting of blood, and venous reservoirs, which are incorporated in the circuit contribute to the inflammatory response [68].

**Table 1:** ECLS modalities

	CPB	VA-ECMO	VV-ECMO	ECCO_2_R
Organ support	Cardiac and pulmonary	Cardiac and pulmonary	Pulmonary: oxygenation and ventilation	Pulmonary: ventilation
Duration	Minutes to hours	Days to weeks	Days to weeks	Days to weeks
Anticoagulation	Very high-dose heparin	Low-dose heparin	Low-dose or no heparin	Low-dose heparin
Reversal	Protamine	No	No	No
Air-blood interface	Yes	No	No	No

### CPB and postoperative pulmonary dysfunction

The impact of CPB on postoperative lung function has been debated. Traditional strategies of no MV during CPB might induce pulmonary dysfunction, due to development of micro-atelectasis, hydrostatic pulmonary edema, and ischemic lung injury secondary to reduction in bronchial artery flow [69]. Furthermore, ECLS-induced inflammation has been associated with pulmonary dysfunction. In adults undergoing CPB, IL-8 levels in the bronchoalveolar lavage were significantly correlated to arterial oxygenation and intrapulmonary shunt at the end of the surgery [70]. Moreover, the length of MV was longer in patients with an exaggerated inflammatory response to CPB [71]. Finally, postoperative concentration of IL-8 was higher in patients ventilated for more than 24 h in comparison to patients ventilated for less than 24 h [72].

Nonetheless, it has been suggested that post-CPB pulmonary dysfunction might be triggered by anesthesia or surgical technique. In patients with good ventricular function and without prior pulmonary diseases, coronary artery bypass on or off pump caused a similar degree of postoperative pulmonary dysfunction [73]. In addition, it has been speculated that the similar left atrium/right atrium ratio of leukocyte count in ventilated and non-ventilated patients during CPB reduces the possibility of the inflammatory response accounting for difference in the incidence of lung injury [74]

## Clinical implications of ECLS-associated inflammatory response

Several studies demonstrated a considerable association between inflammation and outcome during ECLS in its different configurations. In neonates undergoing CPB, Interlukin 6 (IL-6) and IL-8 concentrations correlated with postoperative myocardial dysfunction [75], lactate concentrations, blood product administration, duration of MV, and ICU and hospital length of stay [76, 77]. Moreover, in adults post-cardiac surgery, increased IL-6 levels after CPB were predictive of infection in patients with impaired left ventricular function [78], and preoperative IL-8 concentrations correlated with prolonged postoperative MV [79]. In addition, one specific genetic polymorphism of IL-6 was associated with acute lung injury [80].

During veno-venous extracorporeal membrane oxygenation (VV-ECMO) for severe ARDS, IL-6, IL-8, and tumor necrosis factor-α (TNF-α) levels were associated with an increased risk of in-hospital mortality [25]. IL-6 levels were persistently increased in non-survivors among a mixed group of patients undergoing VV-ECMO and VA-ECMO [81]. Furthermore, higher levels of TNF-α have been correlated with mortality in neonates undergoing VV- or VA-ECMO [23, 52]. Of note, IL-6 was identified as a potentially useful prognostic marker for mortality during ECMO support [81], pulmonary dysfunction after CPB [82], and acute kidney injury after cardiac surgery, both in children [83] and in adults [84].

Greater release of IL-10 after CPB was associated with improved cardiac index and pulmonary gas exchange [85] and increased chance of survival following cardiogenic shock in adults supported with ECMO [86]. Additionally, IL-10 levels in ARDS patients correlated with severity of illness and predicted unsuccessful ECMO weaning and mortality [87].

### Potential therapeutic interventions in ECLS-induced inflammation

A number of different strategies, including pharmacologic agents and non-pharmacologic interventions (i.e., innovative surgical techniques, ECLS circuit modifications, the conduction of anesthesia and ventilation), have been evaluated in experimental [88] and clinical trials [33, 89] with the aim of minimizing the impact of ECLS-related systemic inflammation on patients’ outcome in pediatric [89, 90] and adult patients [91]. However, the impact of these strategies on the post-ECLS clinical course has been conflicting.

Although the administration of steroids during pediatric cardiac surgery has been associated with the attenuation of CPB-induced inflammation [89], their impact on postoperative clinical outcome remains modest [92]. The administration of steroids was associated with a reduction in postoperative infection and duration of postoperative MV and length of stay, but no beneficial effects on mortality and organ complications in adult cardiac surgery patients have been reported [93, 94].

Preoperative statin therapy was associated with a reduction in post-CPB inflammation [95, 96] and an improvement in morbidity and mortality after cardiac surgery [97–99]. However, a recent meta-analysis of randomized control trials (RCTs) found no evidence of benefit for patients’ outcomes [100]. Patients with high preoperative IL-6 levels might be the best candidates for the preemptive administration of statins in cardiac surgery with CPB [101]. Other anti-inflammatory pharmacologic strategies, such as protease inhibitors (i.e., sivelestat, ulinastatin, aprotinin) [102–104] and milrinone [105], have been associated with improved postoperative clinical outcomes, although additional studies are needed to provide a better perspective regarding future applications.

Monoclonal antibodies have been studied as modulators of ECLS-induced inflammation. A novel inhibitory antibody against factor XIIa has been shown to reduced inflammation in ex vivo and animal models of ECMO [106]. Moreover, human monoclonal antibody directed at C5 significantly inhibited neutrophil activation in a model of simulated extracorporeal circuit (ECC) [107]. Mesenchymal stromal cells (MSC) therapy infusion in an animal model of CPB significantly reduced inflammatory cytokines within 3 h and subsequently reduced the harm associated with ischemia-reperfusion injury [108]. Promising results have also been shown with hemoadsorption during CPB [109] and ECMO [110]. However, all these therapeutic options remain experimental.

Many technical modifications of the CPB circuit and surgical procedures were implemented to minimize systemic inflammation secondary to the activation of blood components after contact with the CPB circuit and pulmonary dysfunction after ischemia-reperfusion injury [90, 91]. A minimized extracorporeal circulation system [111] and the circuit coating with poly-2-methoxyethyl acrylate [112] or heparin [113] have been associated with a decrease in the systemic inflammatory response, thus potentially improving organ function after cardiac surgery. By reducing the ischemic insult to the lungs and inflammatory activation, pulmonary perfusion during CPB may decrease systemic inflammatory response and have a protective effect on the lungs [114–117]. However, robust evidence for any beneficial effects is lacking according to a recent meta-analysis [118].

## ECLS as a therapy for systemic inflammation

Despite the fact that different modalities of ECLS have been implicated in driving an intense inflammatory response, ECLS can also be employed to offset it. By replacing the function of the heart or the lung, ECLS may result into a direct reduction of inflammation due for instance to improved perfusion and gas exchange or may allow the reduction of the pro-inflammatory “stress” induced by other life support means, such as MV, with an indirect effect on treating systemic inflammation.

### ARDS, VV-ECMO, and ECCO_2_R

In ARDS, pulmonary and systemic inflammation exacerbated by high MV settings, the so-called ventilator-induced lung injury, can be reduced by a lung-protective ventilation strategy [12], which has been demonstrated to increase patients’ survival [119]. In an interesting recent observational trial, the initiation of VV-ECMO support in mechanically ventilated patients for ARDS was associated with a remarkable decrease in cytokine levels, potentially explained by the achievement of “lung rest” with less alveolar stress and strain [25].

Recently, the hypothesis that the implementation of ultra-protective MV may allow the achievement of minimal alveolar stress and strain, thus further reducing pulmonary and systemic inflammation in ARDS, has been addressed in experimental and observational clinical studies utilizing different ECLS strategies [120–122]. The use of ECCO_2_R has been reported to significantly reduce ARDS patients’ inflammatory response [123, 124]. In an interesting proof-of-concept clinical study, patient with ARDS with high plateau airway pressure despite the delivery of protective MV with tidal volumes of 6 cc/kg of predicted body weight were treated with ECCO_2_R for 3 days in order to further decrease tidal volumes and alveolar distending pressures. ECCO_2_R allowed the tidal volume to be decreased to less than 4 cc/kg of predicted body weight with the consequent significant reduction of the plateau airway pressure, while maintaining normal pH and PaCO_2_. Reduction in the MV intensity resulted in the decrease of alveolar overdistension, as demonstrated by CT scan imaging, and in the significant decreases of the bronchoalveolar inflammatory cytokines IL-6, IL-8, IL-1b, and IL-1Ra [124]. These results were confirmed in a more recent randomized controlled trial that, comparing ultra-protective MV facilitated by ECCO_2_R to conventional lung-protective ventilation, resulted in significant reduction in IL-6 within 24 h of initiation of pumpless arterio-venous ECCO_2_R, but no effect on ventilator-free days or mortality [125]. However, rigorous clinical trials on this topic are needed before this approach can be recommended in clinical practice [126–128].

### Mechanical ventilation during CPB

Although the impact of protective MV during CPB on cytokine levels, pulmonary function, and clinical outcomes is still controversial [129–131], most studies described its beneficial effect on post-CPB systemic inflammatory response [132–135] and lung function [74, 136], thereby potentially improving clinical outcomes [74]. For example, in adult patients undergoing CPB, IL-6 and IL-8 levels in the bronchoalveolar lavage fluid and plasma were higher with high tidal volume/low positive end-expiratory pressure than with low tidal volume/high positive end-expiratory pressure [132]. However, the interesting results of a pilot randomized controlled trial, comparing MV versus no MV during CPB, showed that the group treated with MV had less pulmonary edema and shorter overall duration of MV [74]. It has been proposed that this benefit derives from the partial preservation of bronchial arterial flow. Despite a recent meta-analysis of randomized controlled trials showing that ventilation during CPB may improve post-CPB oxygenation and gas exchange [137], the positive effects of the designated MV techniques are probably short-term and with a questionable impact on the clinical outcome [137, 138].

### VA-ECMO and cardiogenic shock

Institution of ECLS has been associated with reduction inflammation in patients with cardiogenic shock. This was reported to result in hemodynamic improvement in patients with left ventricular assist device used for cardiogenic shock [139]. Furthermore, a significant reduction in the levels of IL-6 and IL-10 was reported in patients following institution of VA-ECMO for post-cardiotomy syndrome and myocarditis [53]. The resultant hemodynamic stability was theorized to lead to improved end-organ perfusion and contributed to recovery from multiple organ failure. Moreover, the use of heparin-coated biocompatible circuits was thought to minimize blood-material interaction and result in reduction of ECMO-induced systemic inflammation.

## Conclusions

Critical illness-associated inflammatory process is complex. It can be secondary to acute illness or due to complex ICU therapeutic interventions. ECLS can induce an inflammatory process that has been associated with morbidity and mortality. On the other hand, it can offer a therapeutic benefit in facilitating lung and cardiac support, which might limit the determinants of inflammation. In the future, the development of progressively more advanced ECLS technology will certainly provide a safer means of advanced life support with potentially higher chances to demonstrate their therapeutic benefit.

## Abbreviations

CPB: Cardiopulmonary bypass
ECC: Extracorporeal circuit
ECCO_2_R: Extracorporeal carbon dioxide removal
ECLS: Extracorporeal life support
ICU: Intensive care unit
IL-10: Interlukin-10
IL-6: Interlukin-6
IL-8: Interleukin-8.
MV: Mechanical ventilation
SIRS: Systemic inflammatory response syndrome
TF: Tissue factor
TNF-α: Tumor necrosis factor-alpha
VA-ECMO: Veno-arterial extracorporeal membrane oxygenation
VV-ECMO: Veno-venous extracorporeal membrane oxygenation
